# Facial aging trajectories: A common shape pattern in male and female faces is disrupted after menopause

**DOI:** 10.1002/ajpa.23878

**Published:** 2019-06-12

**Authors:** Sonja Windhager, Philipp Mitteroecker, Ivana Rupić, Tomislav Lauc, Ozren Polašek, Katrin Schaefer

**Affiliations:** ^1^ Department of Theoretical Biology University of Vienna Austria; ^2^ Dental Clinic Apolonija Zagreb Croatia; ^3^ Study of Anthropology, Faculty of Social Sciences and Humanities University of Zagreb Croatia; ^4^ Faculty of Dental Medicine and Health University of Osijek Croatia; ^5^ Department for Orthodontics, Faculty of Dentistry University of Osijek Croatia; ^6^ Department of Public Health School of Medicine, University of Split Croatia; ^7^ Gen‐info Ltd. Zagreb Croatia; ^8^ Department of Evolutionary Anthropology University of Vienna Austria

**Keywords:** aging, face, geometric morphometrics, menopause

## Abstract

**Objectives:**

Despite variation in lifestyle and environment, first signs of human facial aging show between the ages of 20–30 years. It is a cumulative process of changes in the skin, soft tissue, and skeleton of the face. As quantifications of facial aging in living humans are still scarce, we set out to study age‐related changes in three‐dimensional facial shape using geometric morphometrics.

**Materials and methods:**

We collected surface scans of 88 human faces (aged 26–90 years) from the coastal town Split (Croatia) and neighboring islands. Based on a geometric morphometric analysis of 585 measurement points (landmarks and semilandmarks), we modeled sex‐specific trajectories of average facial aging.

**Results:**

Age‐related facial shape change was similar in both sexes until around age 50, at which time the female aging trajectory turned sharply. The overall magnitude of facial shape change (aging rate) was higher in women than men, especially in early postmenopause. Aging was generally associated with a flatter face, sagged soft tissue (“broken” jawline), deeper nasolabial folds, smaller visible areas of the eyes, thinner lips, and longer nose and ears. In postmenopausal women, facial aging was best predicted by the years since last menstruation and mainly attributable to bone resorption in the mandible.

**Discussion:**

With high spatial and temporal resolution, we were able to extract a shared facial aging pattern in women and men, and its divergence after menopause. This fully quantitative three‐dimensional analysis of human facial aging may not only find applications in forensic and ancient human facial reconstructions, but shall include lifestyle and endocrinological measures, and also reach out to studies of social perception.

## 1 INTRODUCTION

Throughout life, facial shape changes systematically due to growth, maturation, and senescence. What we see on the surface is the joint effect of aging and other processes in several tissue layers. Despite variation in lifestyle and environment, the first signs of facial aging become apparent between the ages of 20 and 30 (Albert, Ricanek Jr., & Patterson, 2007; Windhager & Schaefer, 2016). Facial aging results from cumulative age‐related changes in the skin, soft tissue, and skeleton of the face (Mendelson & Wong, 2012). Its manifestations reflect the combined effects of gravity, facial volume loss, progressive bone resorption, decreased tissue elasticity, and redistribution of fat (Coleman & Grover, 2006). In this article, we focus on age‐related changes in facial shape, leaving aside changes that occur in facial texture, color, and amount of facial hair. Quantifying aging patterns is not only crucial in the fields of facial reconstruction and aesthetic rejuvenation, but is also important in studies of facial recognition as well as interpersonal perception and stereotyping.

**Figure 1:**
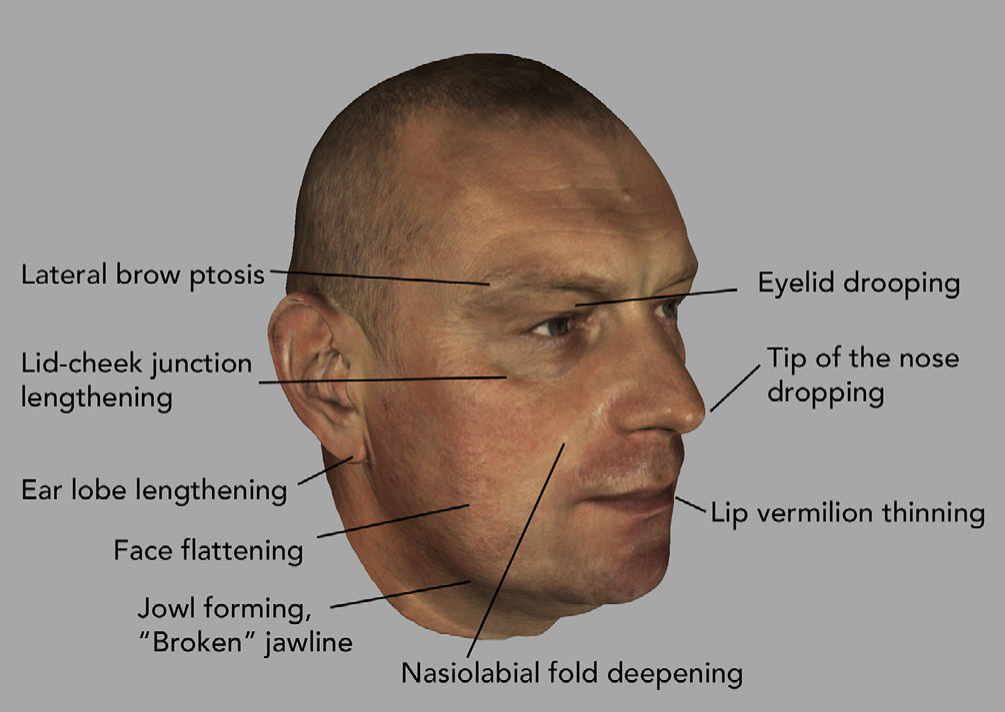
Example facial surface scan with aging‐related features labeled. They are the combined result of skeletal and soft‐tissue alterations and robust age markers in both sexes. Lines and wrinkles are signs of aging too, yet their locations across individual faces are more variable, so that they average out in studies of age‐specific average shapes like ours. Also, they are more susceptible to lifestyle and environmental influences. The 3D model of the example face is publicly available from Artec 3D (https://www.artec3d.com/de/3d‐models/gesichtsscan)

In the centennial anniversary issue of this journal, Bogin, Varea, Hermanussen, and Scheffler (2018) have just updated the Bogin classification system of human life history stages. In our study, we focus on those stages underrepresented in the physical anthropological literature (Ice, 2003): gradual decline (35–50 years), transition/degeneration age (>50 years to senescence), and senescence/old age, which shows variable onset and progression as a function of prior levels of somatic and cognitive reserves. Also, Kirkwood (2017) stresses the variability of aging, caused by “a process of progressive accumulation of defects that stem ultimately from random damage” (p. 1070). Despite individual variation in onset and progression, human facial aging shows a common pattern of morphological, histological, and dermatological changes, as addressed in numerous biomedical studies (Figure 1). Bone tissues along the orbital rim, especially superomedially and inferolaterally, have been shown to recede with increasing age, while the central orbital parts remain relatively stable throughout life (Kahn & Shaw Jr., 2008). This contributes to a more prominent medial fat pad, elevated medial brows, and the typical lengthening of the lid‐cheek junction in older age (Mendelson & Wong, 2012). Retrusion of the bony midface and the maxilla in adds to building and deepening the nasolabial folds and to increasing facial flatness (Pessa et al., 1998; Shaw Jr. & Kahn, 2007). The lengthening of the nose results from an enlargement of the piriform aperture as the bony edges recede, especially in the ascending process of the maxilla. Together with reduced soft‐tissue laxity, this also leads to a drooping nose tip (Rohrich, Hollier Jr., Janis, & Kim, 2004; Shaw Jr. & Kahn, 2007). Moreover, the height and length of the mandible decrease in older ages, whereas the mandibular angle increases (Shaw Jr. et al., 2010). Mendelson and Wong (2012), however, noted that these standard linear measures fail to identify in‐between areas of reduced facial projection, such as the mandible's prejowl region, which becomes more concave with increasing age (Pessa, Slice, Hanz, Broadbent Jr., & Rohrich, 2008; Romo, Yalamanchili, & Sclafani, 2005; Zimbler, Kokoska, & Thomas, 2001).

Collagen fibers are responsible for the resilience and main mass of the dermis. Males have more collagen than females throughout adult life (Shuster, Black, & McVitie, 1975). With increasing age, the amount, quality, and type of collagen change (Galea & Brincat, 2000; Shuster et al., 1975). In both sexes, total skin collagen and skin thickness decrease. Yet, especially after menopause, collagen becomes reduced both in the skin and bone of female faces. Experimental estrogen administration increases skin thickness (as summarized by Brincat, Baron, & Galea, 2005), but mice models indicate that also androgen contributes to the thicker male skin (Markova et al., 2004).

The amount and distribution of subcutaneous fat further contribute to the observable facial shape. This fat is thicker (especially in the medial cheek) and more unevenly distributed in the female than in the male face (Keaney, 2016). With increasing age, however, soft tissue thickness decreases, especially between 20 and 60 years (Wysong, Joseph, Kim, Tang, & Gladstone, 2013). Midfacial ptosis is further enhanced by muscle loss and progressive muscle shortening and straightening (Buchanan & Wulc, 2015). Donofrio (2000) ascribed the physical appearance of tissue sagging to either too little or too much fat (hence the term “sagging paradox”): fat is stored diffusely in young faces, but older faces pocket fat in distinct areas. Such processes also account for ptosis of the brows and eyelid drooping, which already become apparent before age 30 (Zimbler et al., 2001).

Human lips also change throughout adulthood. Dryness increases with age and is higher on the lower lip than on the upper one (Lévêque & Goubanova, 2004). In a qualitative illustration of an aged face, Zimbler et al. (2001) described upper lip flattening and lengthening as well as a thinning and atrophy of the vermilion (red lip). Like the lips, the external ear is built solely from soft tissue. Total ear height increases with age mainly due to lobal height increase in both sexes (Asai, Yoshimura, Nago, & Yamada, 1996; Brucker, Patel, & Sullivan, 2003). Heathcote (1995) reported a lengthening of the ear by 0.22 mm per year in a cross‐sectional study of people aged 30–93 years.

Based on linear measurements of facial photographs of the same person at two ages, Pitanguy et al. (1998) derived a second‐order polynomial model to best fit the ptosis of the midfacial tissues in women with increasing age. Leta, Pamplona, Weber, Conci, and Pitanguy (2000) extended this approach toward lateral views, and both research teams further support most of the above‐described soft‐tissue patterns regarding eyes, lips, and ears in Brazilian patients of European descent. Schmidlin, Steyn, Houlton, and Briers (2018) obtained similar results in African faces and graphed their values in relation to the work of Sforza and colleagues in Italian faces. They confirmed the overall pattern, notwithstanding absolute thickness differences between the populations at a given age stage.

Despite some recent efforts to quantify age‐related shape features of the face beyond single regions (Chen et al., 2015; Mydlová, Dupej, Koudelová, & Velemínská, 2015), the evidence is still largely qualitative for faces of living humans. Combining the scarce quantitative studies is also hindered by the diverse ethnic backgrounds of the participants in these studies (Vashi, Buainain De Castro Maymone, & Kundu, 2016). Therefore, we set out to study age‐related facial shape changes in adults using a geometric morphometric approach. More specifically, we transferred—for the first time—the geometric morphometric toolkit of physical anthropology and its study of growth trajectories (Bulygina, Mitteroecker, & Aiello, 2006; Coquerelle et al., 2011; Mitteroecker, Gunz, Bernhard, Schaefer, & Bookstein, 2004) to human facial aging, including soft tissue. We study changes in appearance with chronological age in a genetically and environmentally homogeneous group from two Croatian islands and a near‐by coastal town, based on three‐dimensional facial surface scans. Local linear regressions allow for an unprecedentedly high temporal and spatial resolution.

## 2 METHODS

### 2.1 Participants

Our sample consists of 32 male (aged 27.7–82.9 years) and 56 female Croatians (aged 26.0–89.8 years) from the coastal town Split and the neighboring islands Korčula and Vis. Demographic, physical, and genetic data were collected as part of the 10,001 Dalmatians project (Croatian national biobank; Rudan et al., 2009). Using a similar sampling regime for participants from Split, Korčula, and Vis (*n* = 2,768), Kolčić et al. (2016) reported a median of 12 years of schooling, more than 50% never smokers, about 25% current smokers, 10% with intense physical activity, and 52–70% with moderate physical activity. The additional analyses of this population indicated unexpectedly high levels of social equality (Smoljanović et al., 2007; Stipčić et al., 2015), which in line with the reduced genetic and environmental diversity made this population very interesting for multifactorial research studies, such as this one.

Faces were surface scanned using an Artec MHT scanner between May 2013 and May 2014. This scanner is characterized by a 3D resolution of 0.5 mm and a 3D point accuracy of 0.1 mm. Color information is captured with 24 bits per pixel. The light source is a flashbulb, so that the participants could face the scanner with eyes open.

Age was computed as the difference between date of birth and date of facial data collection. Twenty‐six women reported that they were postmenopausal and when they had their last menstruation; none of them had hormonal replacement therapy.

### 2.2 Ethical approval and informed consent

This study was approved by the Ethics Committee of the University of Split, School of Medicine (Class 003‐08/11‐03/0005, reg. no.: 2181‐198‐03‐04/10‐11‐0008). Participants from the islands were partly reimbursed their travel costs, while those from the city of Split were not directly reimbursed, but were offered additional health checkups free of charge if they opted to participate in this study. All the methods were carried out in accordance with the relevant guidelines and regulations. Informed consent forms were signed by all participants.

### 2.3 Assessment of facial shape and statistical analysis

**Figure 2:**
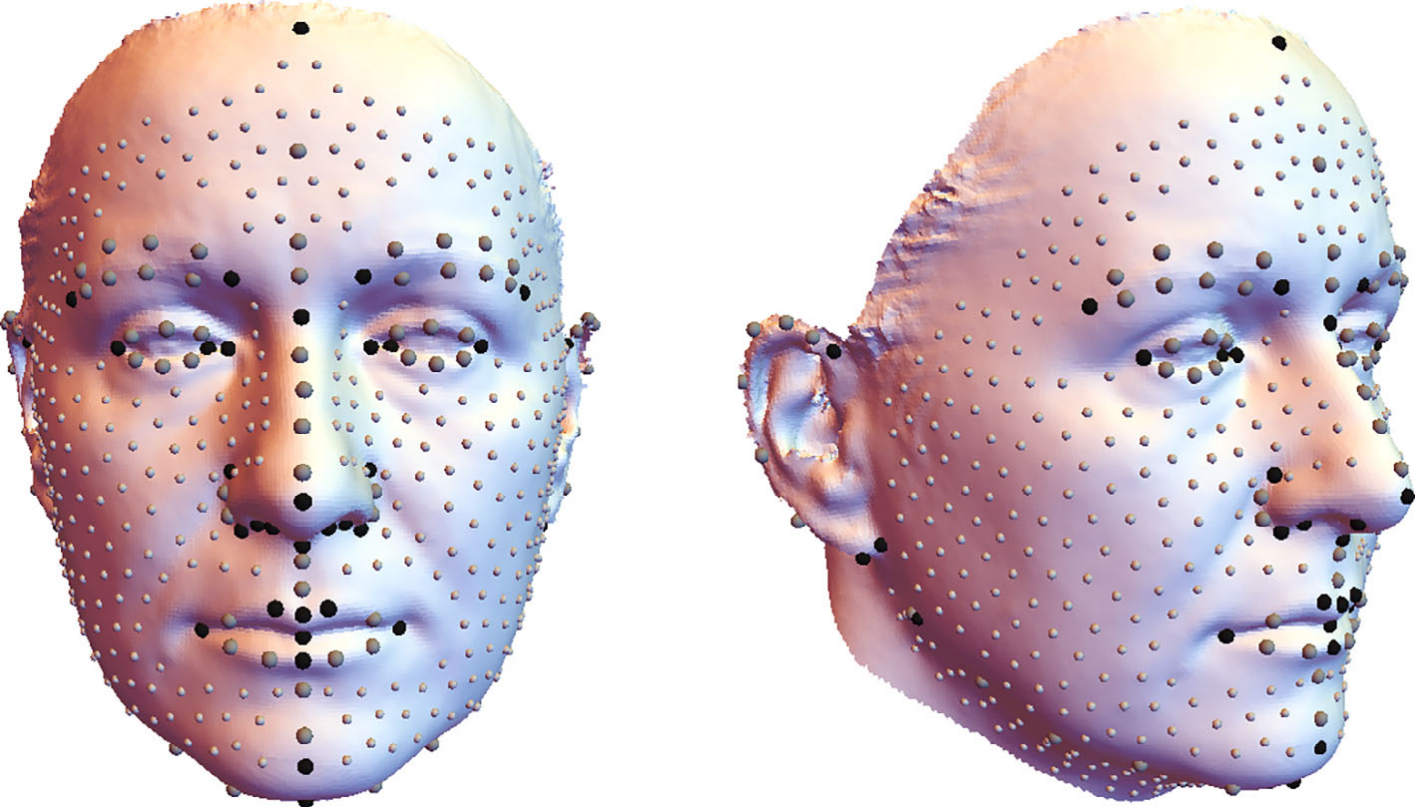
Landmark scheme. Facial surface (sample average) with the 40 fixed landmarks (black spheres), 64 curve semilandmarks (large gray spheres), and 481 surface semilandmarks (small gray spheres) used in the analysis

A set of 40 anatomic landmarks (representing homologous point locations) and 64 curve semilandmarks (representing homologous curves) were manually located on each 3D surface (Table 1, Figure 2). Locating the landmarks was based on local surface curvature and color information. Additionally, a template of 481 surface semilandmarks was digitized on one surface (originating from the extraction of 10 mm equidistant surface points) and then warped and projected onto all other surfaces (Gunz, Mitteroecker, & Bookstein, 2005). Hence, each facial scan was parameterized by 585 (semi)landmarks, 545 of which were allowed to slide along their corresponding curve or surface in order to minimize the bending energy—a measure of local shape difference (Bookstein, 1991)—between each individual and the sample average (Gunz et al., 2005; Gunz & Mitteroecker, 2013). This sliding algorithm establishes geometric homology of the semilandmarks within the sample. In the current application, we modified the standard sliding algorithm by symmetrizing the reference configuration (sample average) in every iteration; this reduces shape asymmetry due to asymmetric spacing of semilandmarks and preserves only the asymmetries that are driven by the actual morphology. After sliding, all configurations were superimposed by Generalized Procrustes Analysis (Mitteroecker, Gunz, Windhager, & Schaefer, 2013; Rohlf & Slice, 1990) and symmetrized by averaging each configuration with its relabeled reflection (Bookstein, 1991; Mardia, Bookstein, & Moreton, 2000).

**Table 1:** Soft‐tissue landmark definitions

Facial landmarks	LM	Definitions fornon‐standard landmarks
**MIDLINE**		
*Trichion*	1	
*Nasion*	2	
*Pronasale*	3	
*Subnasale*	4	
*Labiale superius*	5	
*Stomion*	6	
*Labiale inferius*	7	
*Pogonion*	8	
*Menton*	9	
*Cervical point*	10	The innermost point between the submental area and the neck. Located at intersection of lines drawn tangentially to neck and submental area
Nasal ridge	101–104	Four sliding landmarks between *Nasion* and *Pronasale*
Further midline points		Nine sliding landmarks on midline as the result of surface point extraction
**EYEBROWS**		
*Superciliare mediale*	11[R], 21[L]	
*Superciliare laterale*	12[R], 22[L]	
Lower eyebrow	13–16[R], 23–26[L]	Four sliding landmarks between *Superciliare laterale* and *Superciliare mediale*, placed from lateral to medial along the lower rim of the eyebrow
Upper eyebrow	17–20[R], 27–30[L]	Four sliding landmarks between *Superciliare laterale* and *Superciliare mediale*, placed from medial to lateral along upper rim of eyebrow
**EYES**		
*Endocanthion*	31[R], 40[L]	
*Sclera*	32[R], 41[L]	The most medial point of the visible sclera
*Exocanthion*	33[R], 42[L]	
Lower lid	34–36[R], 43–45[L]	Three sliding landmarks between *Exocanthion* and *Endocanthion*, placed from lateral to medial along rim of lower eyelid
Upper lid	37–39[R], 46–48[L]	Three sliding landmarks between *Endocanthion* and *Exocanthion*, placed from medial to lateral along rim of upper eyelid
**NOSE**		
*Alare superior*	49[R], 55[L]	
Alar curvature	50–51[R], 56–57[L]	Two sliding landmarks between *Alare superior* and *Subalare* on base of nasal ala
*Subalare*	52[R], 58[L]	
*Nostril posterior*	53[R], 59[L]	Most posterior point of nostril margin
*Nostril anterior*	54[R], 60[L]	Most anterior point of nostril margin
**MOUTH**		
*Cheilon*	61[R], 62[L]	
*Crista philter*	63[R], 67[L]	
Upper lip point	64[R], 68[L]	Placed in the middle between *Cheilon* and *Christa philter* along vermilion border
Lower lip points	65–66[R], 69–70[L]	Two sliding landmarks between *Cheilion* and *Labiale inferius* on vermilion border of lower lip
**JAWLINE**		
*Otobasion superius*	71[R], 78[L]	
*Otobasion inferius*	72[R], 79[L]	
Lower jawline	73–77[R], 80–84[L]	Five sliding landmarks between *Otobasion inferius* and *Menton*, placed along lower border of mandible
*Gonion*	99[R], 100[L]	
**EARS**		
External ear	85–90[R], 92–97[L]	Six sliding landmarks on outer border of external ear from *Otobasion superius* to *Subaurale*
*Subaurale*	91[R], 98[L]	

*Note*: Operationalizations of the 40 fixed anthropometric landmarks (italics) and the 64 curve semilandmarks . See Figure 1 for their visualizations on the facial surface. Definitions based on Farkas (1994) as well as Kolar and Salter (1997). Curve semilandmarks were placed equally spaced between the two specified fixed landmarks.

Abbreviations: L, on left side of face; LM, landmark number; R, on right side of face.

For both sexes, we estimated age‐related shape changes by local linear regressions in a moving window of 23 individuals. The sequences of age‐ and sex‐specific average shapes, as predicted by the local regressions, were interpreted as the male and female trajectories of average facial aging. We computed a principal component analysis of these age‐specific average shapes and projected the individual face shapes into this principal component space. Similar to a between‐group PCA, this ordination is optimized to represent sex‐ and age‐dependent shape differences (Mitteroecker & Bookstein, 2011). Shape trajectories were visualized by surface morphs, computed by warping (Thin‐Plate Spline interpolation; Bookstein, 1989) the vertices of one facial surface (roughly 83,000 points) to the target configurations based on the 585 landmarks and semilandmarks.

In addition to the mean shapes, we also estimated the *rate* of facial aging within the sampled age period, separately for both sexes. Within the moving window of 23 individuals, we performed a multivariate regression of facial shape on age and computed the two norms of the resulting vector of regression coefficients. This quantity corresponds to the average amount of facial shape change (in units of Procrustes distance) per year. All analyses and their visualizations were performed using Mathematica 11.

### 2.4 Data availability statement

The data sets generated during and/or analyzed during the current study are available from the corresponding author on reasonable request.

## 3 RESULTS

**Figure 3:**
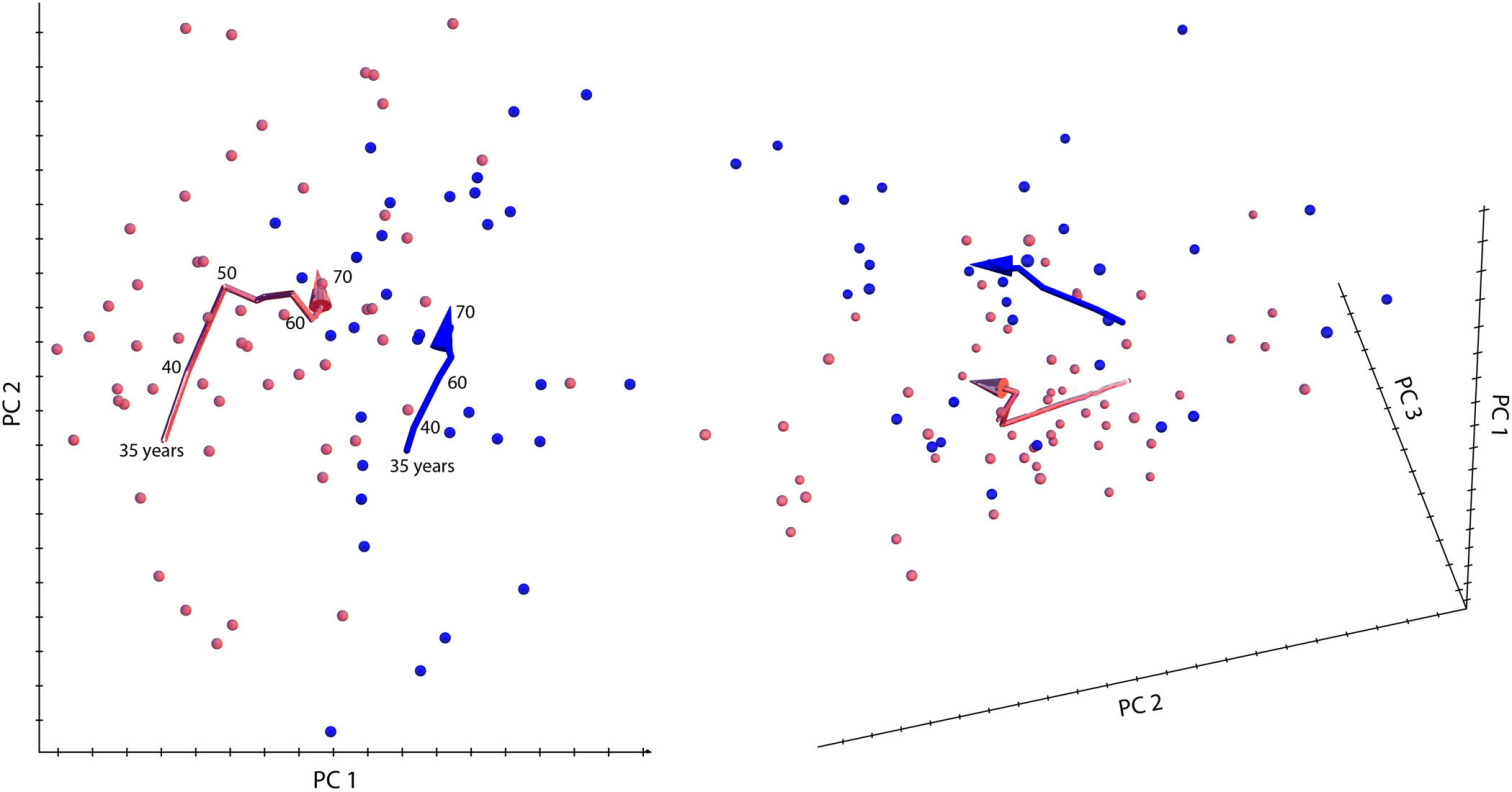
Male and female trajectories of average facial aging as predicted by local linear regressions. The first three principal components (PCs) accounted for 92% of the variation among age‐specific mean shapes. Within the first two PCs, the male aging trajectory (blue) was roughly linear and parallel to the first segment of the likewise linear female trajectory (red). Yet, their direction differed somewhat along PC 3. After 50 years of age, the female aging trajectory turned sharply, indicating a different facial aging pattern in postmenopausal women

**Figure 4:**
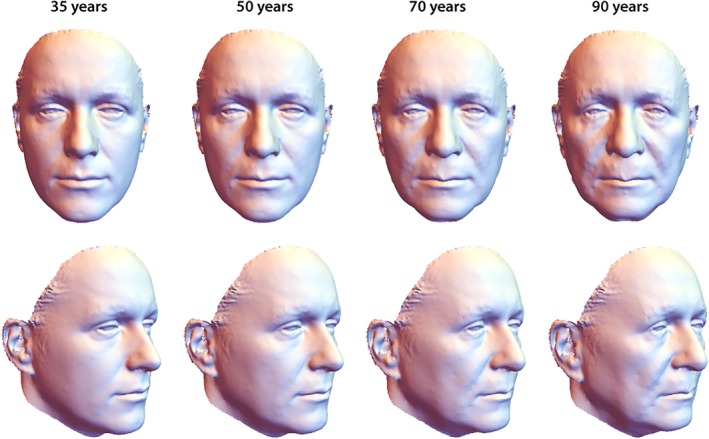
Visualization of male facial aging, derived from a linear regression of shape on age (*p* < .001, *n* = 32). The predicted 90‐year old face extrapolates the actual age range sampled. With increasing age, the face flattened, jowls formed, nasal tips dropped, eyelids drooped, and lips thinned

**Figure 5:**
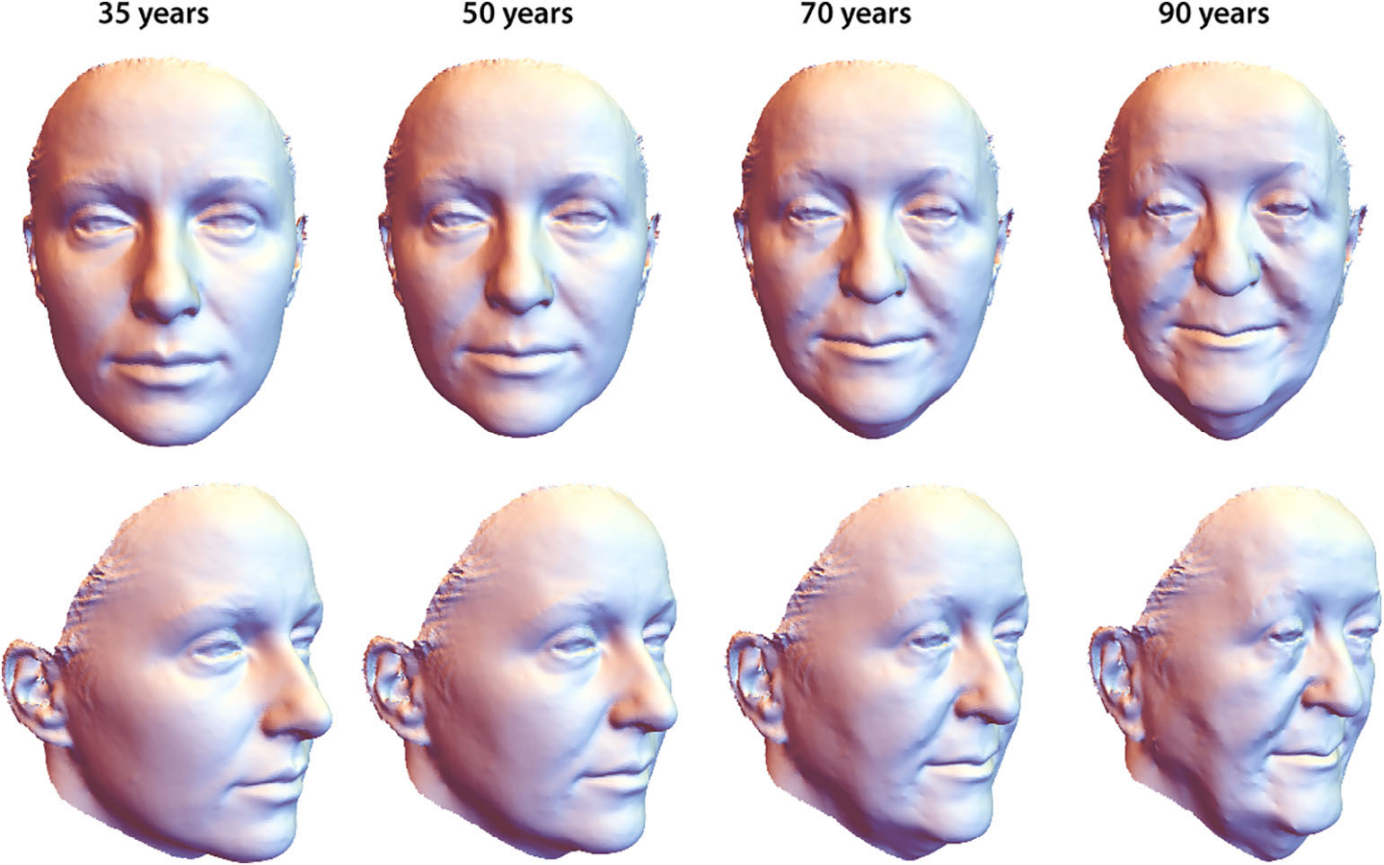
Visualization of female facial aging in women younger than 55 years (first segment of the trajectory depicted in Figure 3), derived from a linear regression of face shape on age in women aged ≤54 years (*p* < .0001, *n* = 23). Broadly, the predicted 70‐year and 90‐year faces are extrapolations of premenopausal aging to depict the shape changes more legibly. Aging in younger female faces resembled the male pattern, yet at about twice its pace

**Figure 6:**
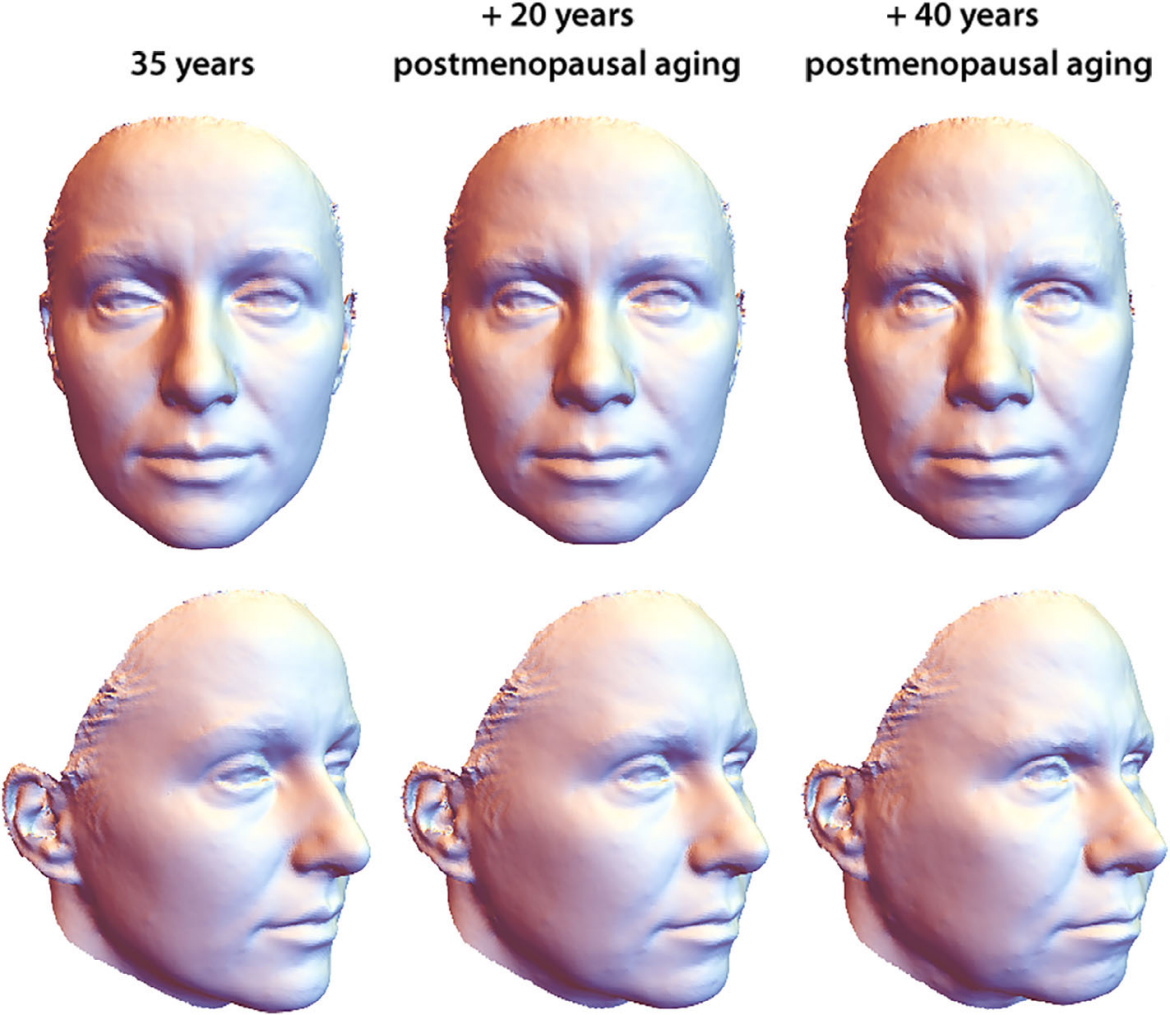
Visualization of postmenopausal female facial aging, derived from a linear regression of facial shape on the time since the final menstrual period (*p* < .0001, *n* = 26). For comparison, this postmenopausal aging pattern is applied to the 35‐year female mean shape, as in Figure 5. This visualization contrasts premenopausal and postmenopausal aging but, of course, does not yield realistic face shapes. Postmenopausal female aging mainly reveals a reduction in the relative size of the lower face, particularly of the chin

The first three principal components (PCs) accounted for 92% of the variation among age‐specific mean shapes. Despite strong variation in individual face shape, PC 1 discriminated male and female configurations well. Within the first two PCs, the male aging trajectory was approximately linear and parallel to the first segment of the female trajectory, which extended approximately linearly from 35 to 50 years of age (even though the two differ somewhat in direction along PC 3; Figure 3). The linearity of the trajectories indicates constant patterns of facial shape change in the assessed age interval (Huttegger & Mitteroecker, 2011). In both sexes, increasing age was associated with a flattening of the face as well as an overall sagging of soft tissue resulting in a “broken” jawline, deepened nasolabial folds, and smaller visible areas of the eyes. Further characteristics of advanced age were relatively thin lips, a drooping tip of the nose, and lengthened ears (Figures 4 and 5). The linear regression of face shape on age in males was statistically significant (*p* < .001) and accounted for 7% of total shape variation and for 38% of the variance in the regression score (projection of shape vectors on the vector of regression coefficients). The female trajectory consisted of two approximately linear segments, separated by a sharp bend at about 50 years of age. The linear regression of face shape on age in females younger than 55 years accounted for 15% of total shape variance and for 40% of the variance in the regression score (*p* < .0001). Thereafter, however, the female aging trajectory changed starkly in direction, indicating a different facial aging pattern (Figure 3). The regression of face shape on age in women beyond 50 years of age had little explanatory power (2% explained variance) and was not statistically significant. This is also reflected by the somewhat unstable local linear regressions in the second half of the female trajectory (Figure 3). In contrast to chronological age, however, the time since the last menstruation predicted 5% of total facial shape variance in postmenopausal women and 35% of the regression scores (*p* < .0001). This pattern of postmenopausal female aging mainly consisted of a reduced mandibular size, particularly the chin (Figure 6), and of some soft‐tissue sagging in the orbital region and along the jaw line.

**Figure 7:**
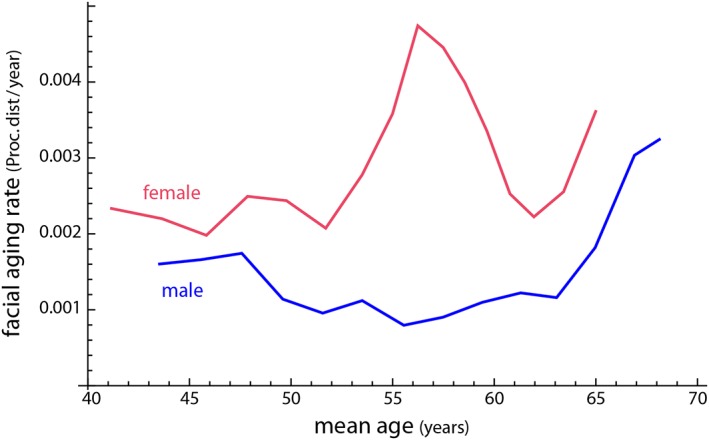
Rates of male and female facial aging, estimated as the average amount of facial shape change in Procrustes distance per year. Female faces change faster than male ones, up to three times as fast at a speed peak between the ages of 50–60 years

The female rate of facial aging (the average amount of shape change per year) is higher than the male rate (Figure 7). Before age 50 and also after age 60, female faces age—on average—about twice as fast as male faces; between 50 and 60 years, this sex difference in aging rate is even more pronounced (up to three times faster).

## 4 DISCUSSION

Whereas previous aging studies relied on largely qualitative descriptions or single linear or angular measures (Mendelson & Wong, 2012), we present a fully quantitative three‐dimensional analysis of facial aging. We demonstrated a shared average facial aging pattern in men and premenopausal women, but an average pace of aging twice as high in females than in males. This shared pattern of facial aging comprises several local shape changes (e.g., relatively smaller eyes, thinner lips) and a widened lower face (due to sagging soft tissue; Figures 4 and 5). Our findings resemble the 3D visualizations of facial aging from Chinese and Czech samples (Chen et al., 2015; Mydlová et al., 2015) as well as the qualitative and quantitative descriptions of single regions as reviewed in the introductory paragraphs. However, our results do not seem to confirm a decreasing mid‐facial width‐to‐height ratio as claimed by Hehman, Leitner, and Freeman (2014).

For the first time, we depicted the stark and relatively sudden divergence of postmenopausal from premenopausal female facial aging. While chronological age then no longer significantly predicted facial shape, menopausal years (amount of time since last menstruation) did. A single linear shape regression across the whole female sample, as performed in earlier studies, would have overlooked this signal; only the use of local regressions made it possible to detect the nonlinear aging pattern in females. After menopause, women show a stronger reduction in the jaw area, particularly in the chin, compared with men. This sex difference presumably results from the various hormonal changes during menopause and andropause, which are dominated by the estrogen reduction in females and the testosterone reduction in males. Both hormones are known to affect bone metabolism, but bone resorption is primarily controlled by estrogen in both sexes (Clarke & Khosla, 2010; Falahati‐Nini et al., 2000; Khosla, Melton, & Riggs, 2001). Estrogen loss also affects the jawbones (Ejiri et al., 2008; Mutneja, Dhawan, Raina, & Sharma, 2012). The much larger reduction of estrogen in women than in men may thus cause the more pronounced female bone resorption. Estrogen loss also seems to be closely linked to sagging skin, reduced skin thickness, and other degenerative changes of dermal elastic tissue due to the loss of the underlying collagen (Lephart, 2018; Tobin, 2017).

Our findings also add to Shaw et al.'s report (2010) of an increase in the mandibular angle and a decrease in mandibular height and length in both sexes, with the strongest effects in female postmenopausal years. These results, along with the significant association of facial shape with years since last menstruation, suggest that variation in the age at menopause contributes to individual variation in facial appearance and aging. Similarly, Chen et al. (2015) found that Chinese people of the same chronological age differed by about plus or minus 6 years in terms of “facial age” (i.e., the age predicted from facial morphology) and that the amount of deviation increases after age 40. Further support comes from reported associations of collagen loss with the number of postmenopausal years, not chronological age (reviewed in Tobin, 2017). Despite considerable individual variation in age at menopause, we found that the *average* trajectory of female facial aging bends at approximately 50 years (Figure 3), which fits well with published menopausal data. In a meta‐analysis on age at natural menopause, European countries showed an average age of 50.5 years (Schoenaker, Jackson, Rowlands, & Mishra, 2014).

Independent of changes in aging pattern, we found that the *rate* of aging (the average magnitude of facial shape change, regardless of the pattern of shape change) was relatively constant in males and increased only after 60–65 years. Women generally had a faster average rate of facial aging than men, but it started to increase considerably at about 52–55 years; it decreased from 57 to 60 years and increased again thereafter. The temporal pattern fits well to findings on hormone changes in a longitudinal study by Randolph et al. (2011). They reported a decrease of serum estradiol concentration 2 years before the final menstrual period and a maximal rate of change around the final menstrual period. After another 2 years, the concentration stabilizes at about one third of the level before the decrease. Brincat et al. (2005) also concluded, based on a wide literature review, that 30% of skin collagen is lost in the first five postmenopausal years, with an average decline of 2 % per year over a period of 15 years. Similarly, using methylation levels as epigenetic aging markers, Levine and colleagues (2016) found that menopause accelerates biological aging.

At first glance, it seems puzzling to find higher facial aging rates in women who, on average, live longer. This paradox has also been noted by Finkel, Sternäng, and Wahlin (2017) for functional biological aging trajectories based on body measures such as muscle strength, gait, lung functioning, and sensory abilities. The divergence of male and female mortality rates is quantified by dividing male by female mortality rate. Kruger and Nesse (2006) calculated this ratio for Croatian data from 1998 to 2002, and found higher male mortality rates for all age categories, which holds true cross‐nationally (Kruger & Nesse, 2004). Part of this is explained by behavioral differences that can be attributed to larger male reproductive variation and the resulting evolutionarily shaped preference for risk taking. Along these lines, higher rates of health‐adverse behaviors, such as working in hazardous occupations, smoking and drinking (liver disease and cirrhosis), contribute to excess male mortality in older ages, while accidents, homicides, and suicides predominate in young adult ages. Also, the epidemic of coronary heart disease in industrialized countries following several decades after increased consumption of dietary fats seems to have a greater toll on males than females (Lawlor, Ebrahim, & Davey Smith, 2001), accounting for 26% of male excess life years lost in the United States (Kruger & Nesse, 2004). Other lines of research see a causal relationship of increased male morality with sex chromosomes (XY, across species; Marais et al., 2018 for a review). The amount of effect of testosterone‐mediated immunosuppression in populations where parasite burden is low, however, remains controversial (Regan & Partridge, 2013).

Due to our sample structure, we did not measure individual aging sensu stricto, but estimated the sex‐specific average patterns of facial aging in this population. We could thus not assess the association of facial aging with individual lifestyle parameters, endocrine status, or molecular aging markers (Krištić et al., 2014). Future studies may also include color information (Mayer, Windhager, Schaefer, & Mitteroecker, 2017), which can be helpful in interpreting health problems related to facial aging. Nonetheless, the *average* facial aging pattern is most relevant to aid forensic case identification, where physiological and lifestyle data usually are unavailable. It would be useful, however, to study within and between population variation in facial aging as well as its association with environmental factors, such as temperature and annual hours of sunshine (cf. the extreme case of a truck driver's face showing unilateral photoaging, Gordon & Brieva, 2012). For instance, African faces tend to have thicker facial tissue than any other group, and Japanese faces have thinner tissues than most other groups except for the side of the head (Wilkinson, 2004). In their study of adult African American and European American dry crania, Williams and Slice (2010) identified several two‐ and three‐way interactions among age, sex, and ancestry, indicating that cranial aging differs between sex and ancestry groups, specifically in the curvatures of the orbits, zygomatic arches, and maxillary alveolar process.

The region of the Eastern Adriatic in Croatia represents a well characterized “genetic isolate” population (Rudan, Campbell, & Rudan, 1999): Its comparably homogeneous nature of the communities with reduced diversity of exposure to environmental factors and of genetic heterogeneity, makes our specific cross‐sectional design an appropriate proxy for longitudinal data until they become available. Along these lines, we expect the threshold for detecting patterns due to biological causes of facial shape variation (such as aging) to be lower than in a more heterogenous reference population. However, based on population history (Rudan, 2006), there is no reason to assume that the aging pattern presented, will not generalize to other populations of European descent. But as, to our knowledge, there are no rigorously quantitative studies of facial aging like our's, subsequent analyses may focus on this question in greater detail, allowing a comparative claim that is rooted in high‐quality data.

Our geometric morphometric approach to study facial aging may contribute to facial imaging and reconstruction in forensic anthropology meeting the increasing demands for scientific and statistical rigor in case analysis and reporting (Algee‐Hewitt, Kim, & Hughes, 2018). Current methods using facial images to facilitate human identification include facial approximation and photographic superimposition, face progression, facial depiction, face mapping, automated facial recognition systems, and newly emerging methods of so‐called “molecular photofitting” (Stephan, Caple, Guyomarc'h, & Claes, 2019). Reproducing an individual's face from skeletal remains is not just part of forensic facial reconstruction (Wilkinson, 2004), but also used in paleontological and archeological facial depiction for people from the past (Wilkinson, 2018), which until recently relied largely on poorly verified recommendations to create faces (Hayes, 2016). Some quantitative approaches do not explicitly represent facial aging: For instance, in their landmark paper on modeling facial appearance from DNA, Claes et al. (2014) limited the age of their participants to the ages between 18 and 40 to “minimize age‐related variation in facial morphology” (p. 10). Futures studies building on our line of research could help overcoming such limitations and contribute to a better estimation of facial appearance, especially when age estimation is supported by multiple sources.

Understanding age‐related changes in the face is also of large societal and economic interest in modern societies. The Cosmetic Surgery National Data Bank Statistics (2018) of the American Society for Aesthetic Plastic Surgery for 2017 reports 123,000 procedures of eyelid surgery in women and 22,300 in men. In addition, in both sexes combined, there were 82,400 surgical face‐lifts, 22,700 brow lifts, 6,000 chin augmentations, 10,500 ear surgeries, 54,700 face‐related fat transfers, 28,500 neck lifts, and 38,700 procedures of nose surgery, along with many more nonsurgical procedures. Most of these categories increase in number every year. About 50 % of the total number of eyelid surgeries and facelifts were carried out in persons aged 51–64 years, and another roughly 30 % in individuals older than 65 years.

Enhanced longevity is a main contributor to projected gains in life expectancies at birth (Kontis et al., 2017), which doubled across industrialized countries over the last 200 years. This emphasizes the importance of understanding age‐dependent changes in facial morphology in older age together with their social perception.

## CONFLICT OF INTEREST

The authors declare no potential conflict of interest.

## AUTHOR CONTRIBUTIONS

T.L. and K.S. conceived of the study. T.L. and O.P. were responsible for questionnaire design, data collection as well as participant interactions conducted with I.R.; S.W. and K.S. provided the surface scan protocol. S.W., P.M., and K.S. designed the landmark scheme and supervised landmark digitization. S.W. prepared the data set, and P.M. conducted the analysis and created the figures. S.W., P.M., and K.S. wrote the main manuscript text. All authors reviewed the manuscript.
